# Development and Application of Novel Chemiluminescence Immunoassays for Highly Sensitive Detection of *Anisakis simplex* Proteins in Thermally Processed Seafood

**DOI:** 10.3390/pathogens9100777

**Published:** 2020-09-23

**Authors:** Maciej Kochanowski, Mirosław Różycki, Joanna Dąbrowska, Jacek Karamon, Jacek Sroka, Ewelina Antolak, Aneta Bełcik, Tomasz Cencek

**Affiliations:** Department of Parasitology and Invasive Diseases, National Veterinary Research Institute, 24-100 Puławy, Poland; mrozycki@piwet.pulawy.pl (M.R.); joanna.dabrowska@piwet.pulawy.pl (J.D.); j.karamon@piwet.pulawy.pl (J.K.); jacek.sroka@piwet.pulawy.pl (J.S.); Ewelina.Antolak@piwet.pulawy.pl (E.A.); aneta.belcik@piwet.pulawy.pl (A.B.); tcencek@piwet.pulawy.pl (T.C.)

**Keywords:** *Anisakis simplex*, immunoassay, chemiluminescence, sandwich ELISA, competitive ELISA, seafood, fish product, food-borne parasite, fish-borne nematode

## Abstract

The third-stage larvae (L3) of *Anisakis simplex* are the most important source of hidden allergens in seafood products. However, there exist no commercial methods for detecting *Anisakis* proteins in food. Furthermore, only a few methods have been validated for the detection of *A. simplex* in thermally processed food. The aims of our study are (i) the development and validation of high-sensitivity chemiluminescent (CL) immunoassays for the detection of *A. simplex* proteins in processed seafood, (ii) and *A. simplex* antigen detection in common seafood products from Polish markets. We developed and validated CL sandwich ELISA (S-ELISA) and CL competitive ELISA (C-ELISA) methods for *A. simplex* proteins detection in food, with respective detection limits of 0.5 and 5 ng/mL. The usefulness of the assays for detecting *A. simplex* proteins in highly processed food was evaluated by examination of autoclaved canned fish spiked with *A. simplex* larvae (1–8 larvae/200 g). Commercial real-time PCR was unable to detect *A. simplex* in autoclaved samples at all levels of enrichment with *Anisakis* larvae. CL-S-ELISA was used to test various types of seafood products from Polish markets. Among all tested products (*n* = 259), 28% were positive. *A. simplex* antigens were found mostly (*n* = 39) in smoked fish products: mackerel, herring, cod, and hake. Other positive samples were found in marinated herrings, canned cod livers, canned mackerels, and surimi sticks. In tuna, Atlantic argentine, anchovy, sardine, sprat, and squid products, *A. simplex* antigens were not detected. This study provides novel effective tools for the detection of *A. simplex* proteins in processed food and highlights the potential allergic hazards for *Anisakis*-sensitized Polish consumers of seafood.

## 1. Introduction

*Anisakis simplex* is a pathogenic nematode belonging to the genus *Anisakis*, family Anisakidae, and order Ascaridida. The final hosts of this pathogen are marine mammals, intermediate hosts are crustaceans, while marine fish and cephalopods are intermediate and/or paratenic hosts [1]. *A. simplex* is a nematode widespread in many different species of marine organisms, mainly in the Arctic-Boreal region [1,2,3,4,5,6]. Humans can become accidental hosts as a result of the consumption of live third-stage larvae in seafood dishes. In humans, this disease is called anisakiasis, which mainly affects the gastrointestinal tract (primarily the stomach and intestines), where larvae penetrate the mucosa and submucosa causing their damage.

In addition to the pathogenic effect associated with tissue damage, *Anisakis* larvae can cause allergic reactions in sensitized humans. Furthermore, many *Anisakis* allergens are resistant to low or high temperatures and pepsin digestion; therefore, dead larvae—even those subjected to intensive technological processes such as autoclaving—may cause hypersensitivity reactions [7]. Cases of *Anisakis*-induced hypersensitivity by contact with and inhalation of allergens have also been reported [8]. The worldwide prevalence of hypersensitivity to *Anisakis* is difficult to estimate, as it varies according to the geographical area, characteristics of the population studied, diagnostic criteria, and laboratory assays [9]. It is considered that, in endemic countries, the number of highly sensitized humans in the general population could be approximately 7%.

To prevent anisakiasis, many countries, such as countries in the European Union (EU), United States of America (USA), and Canada, require the freezing of raw fish before placing on the market or other processing to kill viable parasite larvae [10]. However, it should be emphasized that, according to the published studies, single *A. simplex* larvae can survive at −20 °C for 24 h [10] and, according to other studies, even up to 60 h [11]. Interestingly, Sanchez-Alonso et al. [12] showed that *A. simplex* L3 larvae during freezing release more allergens than the larvae not exposed to low temperatures, which can increase the allergic potential of frozen seafood containing *Anisakis*.

According to regulations in many countries, including countries of the EU, fish products need to be tested for the detection of parasites [13,14]. The most common techniques used for this purpose are visual inspection, pressing, and digestion methods. However, these techniques are not suitable for the examination of highly processed products in which *A. simplex* larvae have disintegrated and cannot be morphologically identified. Therefore, the examination of highly processed seafood products, such as pates, salads, canned food, paste, surimi, etc., is extremely rarely carried out, and most studies have focused on testing fish or slightly processed seafood. Immunoassays and molecular methods may be used to detect the presence of *Anisakis* in processed seafood products. However, the results of molecular tests are not correlated with the occurrence of allergens which pose the greatest risk to consumers of processed food [15,16]. In addition, PCR methods are less effective when testing highly processed foods and products with high protein abundance [17,18]. In such cases, PCR methods should be used as an additional test to improve the reliability of immunoassays [19]. Unfortunately, immunoassays for the detection of *Anisakis* spp. in food are not commercially available and laboratories testing seafood have been forced to develop in-house assays.

Chemiluminescence-based detection allows for an increase the sensitivity of immunoassays by at least two orders of magnitude, compared to colorimetric detection [20]. There currently exist many commercially available enhanced chemiluminescence substrates which allow for more sensitive detection, such as the new generation of 1,2-dioxetane compounds. Food products are a very diverse group which often present a difficult matrix to detect pathogens in. Hence, sensitive chemiluminescence tests have found a particular application in their examination.

In this study, we developed 1,2-dioxetane-based chemiluminescence (CL) competitive ELISA (C-ELISA) and sandwich ELISA (S-ELISA) methods for the sensitive detection of *A. simplex* proteins in highly processed seafood. The performance of both assays was evaluated using spiked thermally processed samples. CL-S-ELISA was also applied to survey different seafood products from the Polish market.

## 2. Results

### 2.1. SDS-PAGE and WB Analysis of A. simplex Antigens

**Figure 1:**
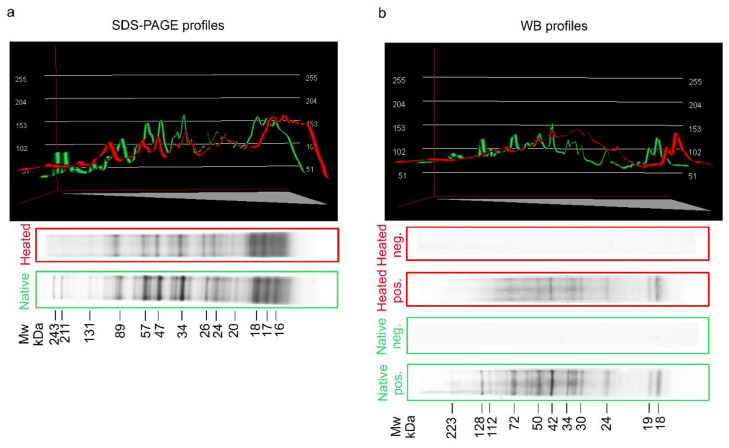
Colloidal Coomassie-stained 4–20% SDS-PAGE analysis of native and heated (for 60 min at 100 °C) crude (CR) antigen of *A. simplex*. (**a**). Western blot (WB) reactivity of anti-*A. simplex* rabbit IgG antibodies with native and heated (for 60 min at 100 °C) CR antigens of *A. simplex*. (**b**). Molecular weight (Mw) estimations in kilodaltons (kDa), densitometric plots of SDS-PAGE and WB profiles were performed using the Bio-1D software (Vilber Lourmat, ver. 15.07, Marne-la-Vallée, France). Green lines in the plots show profiles of the native antigen, while red lines show profiles of the heated antigen. Pos. -strips incubated with hyperimmune serum from a rabbit immunised with native *A. simplex* CR antigen; Neg. -strips incubated with rabbit pre-immune serum.

Figure 1a shows the SDS-PAGE multiband profiles of native and heated (for 60 min at 100 °C) crude (CR) antigens of *A. simplex*. The band profile of the heated antigen was very similar to that of the native antigen. Only a few bands with high molecular weights ranging from 132 to 244 kDa which were found in the native antigen did not appear in the heated antigen. The band intensities of the heated antigen were also slightly lower compared to the native.

WB reactivity profiles of anti-*A. simplex* rabbit IgG antibodies with both *Anisakis* antigens are presented in Figure 1b. The major WB bands of native antigen were visible at the following molecular weights: 18, 19, 24, 30–34, 42, 50–60, 72, 112, 128, and 223 kDa. The profile of the antigen heated at 100 °C was consistent with that of the native antigen; however, similar to the SDS-PAGE profiles, the number and intensities of bands were slightly reduced. The major WB bands of heated antigen were observed at the following molecular weights: 18, 19, 24, 30–34, 42, 50, and 72 kDa. The background in the WB profile of the heated *A. simplex* antigen was higher than in the native antigen.

Densitometric measurements of SDS-PAGE and WB profiles for both antigens are shown in Table 1. Parameter values of the native and heated antigens were similar in SDS-PAGE profiles as well as WB profiles. However, small differences were found between minimum gray values. The minimum gray value of SDS-PAGE profiles was 56 for the native antigen and 102 for the heated antigen, while the minimum gray value of WB profiles was 74 and 131 for native and heated antigens, respectively. The standard deviation (SD) of the gray value of SDS-PAGE was slightly higher in native (42) than heated antigens (35.23). Similarly, the SD of the gray value of native antigen (34.69) was slightly higher than in heated antigen (29.27) in WB profiles. The values of the other densitometric measurements—mean gray value, maximum gray value, and integrated density—were very similar between native and heated antigens.

### 2.2. Analytical Performances of Assays

**Figure 2:**
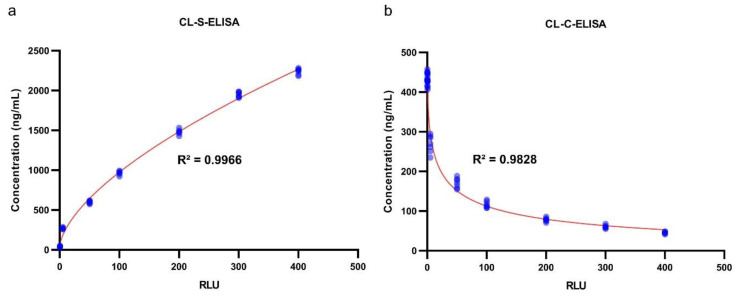
Calibration curves of CL-S-ELISA (**a**) and CL-C-ELISA (**b**). Samples used to generate calibration curves (seven replicates at each level of *A. simplex* antigen concentration, *n* = 63) were heated for 60 min at 100 °C. The concentration (in ng/mL) of *Anisakis* antigen used for spiking trout samples versus Relative Light Units (RLU) observed in assays were plotted using the GraphPad Prism software (GraphPad Software, ver. 8.3.1, La Jolla, CA, USA). Four-parameter logistic regression was used for curve fitting and calculating the coefficient of determination (R^2^) using the GraphPad Prism software. Red lines show fitted curves and blue points show the results of individual samples.

**Figure 3:**
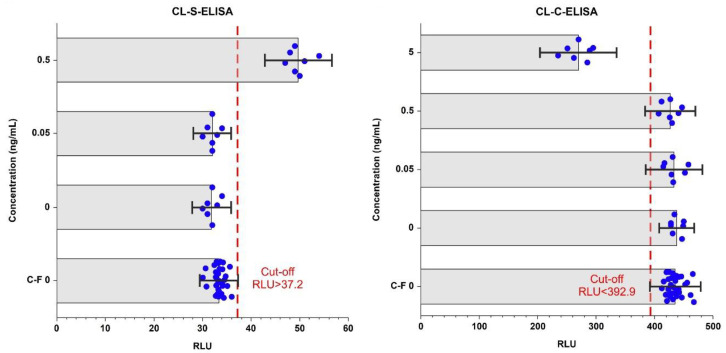
The limit of detection (LOD) and cut-off values of CL-S-ELISA (**a**) and CL-C-ELISA (**b**). The bar chart shows the average Relative Light Units (RLU) observed in assays against the concentration of *A. simplex* antigen (in ng/mL). In this figure, the results for samples used for the cut-off calculation (C-F 0; 30 replicates) and generation of the calibration curve (seven replicates at each level of spiking) are shown. The cut-off values were calculated as average RLU +/− 3 standard deviations (SD) of the raw and processed trout samples. Error bars shown are three times SD. Blue dots display the results of individual samples and red dashed lines show the cut-offs.

**Figure 4:**
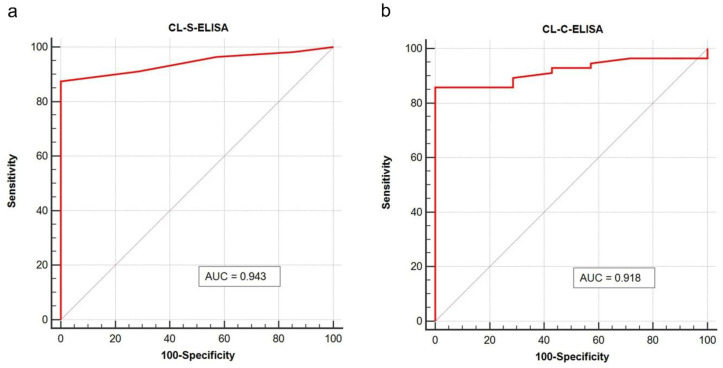
Receiver operating characteristic curve (ROC) plots with calculated Area Under the Curve (AUC) of CL-S-ELISA (**a**) and CL-C-ELISA (**b**). Trout samples (heated extracts spiked with *A. simplex* antigen) used to validate assays (*n* = 63) were subjected to this analysis. The Medcalc software (MedCalc Software Ltd., ver. 19.3, Mariakerke, Belgium) was used to performed calculations and plotting.

The analytical performances of the CL-ELISA methods were evaluated using heated (at 100 °C for 60 min) trout samples spiked with *A. simplex* antigen, as well as non-spiked samples (see Section 4.8.1). No cross-reactions with raw muscle tissue of different fish species and differently processed trout samples were found in both assays (see Section 4.10.5). The estimated limit of detection and precision values of the CL-ELISA methods are presented in Table 2, whereas the calibration curves of the assays are shown in Figure 2. Cut-off values for both CL-ELISA methods were calculated as the mean of Relative Light Units (RLU) of the non-spiked with *Anisakis* raw and processed trout samples plus three times the standard deviation (SD; see Section 4.10.4). Cut-off values are displayed in Figure 3. The appropriateness of the selected cut-off values was confirmed using receiver operator characteristic (ROC) curve analyses (see Figure 4 and File S1). No cross-reactivity with the muscle tissue of different raw fish species and processed fish products was found in either assay. The limit of detection (LOD) of CL-S-ELISA (0.5 ng/mL) was 10 times better than that of CL-C-ELISA (5 ng/mL). CL-S-ELISA had lower intra- and inter-assay variations (better precision), compared to CL-C-ELISA. Furthermore, the standard curve of CL-S-ELISA was slightly better fitted to the four-parameter logistic regression model than that of CL-C-ELISA. The calculated area under the ROC curve (AUC) values were high for both assays, indicating that both CL-ELISA methods were highly accurate; however, the AUC of CL-S-ELISA was slightly higher (see Figure 4). The performance of CL-S-ELISA was also statistically significantly better than that of CL-C-ELISA (McNemar *χ*^2^ test, *p* = 0.0156).

### 2.3. Effectiveness of CL-ELISAs and Commercial Real-Time PCR for the Examination of Autoclaved Canned Food

**Figure 5:**
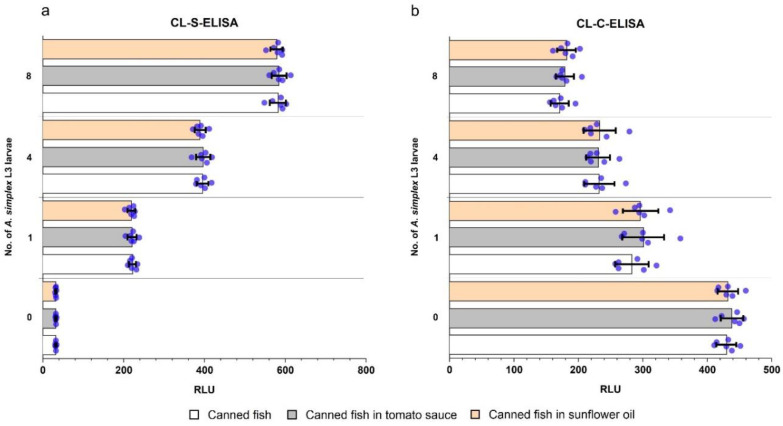
Examination of autoclaved (for 60 min at 121 °C) canned fish products using CL-S-ELISA. (**a**) and CL-C-ELISA (**b**). Three different types of products were tested: canned fish, canned fish in tomato sauce, and canned fish in sunflower oil. Samples were spiked with the following numbers of *A. simplex* L3 larvae: 1, 4, and 8. Non-spiked products were prepared as well. Six replicates of these samples were examined. The bar chart shows the average Relative Light Units (RLU) observed in assays. The bar errors show standard deviations and blue dots display the results of individual samples.

Results of the examination of autoclaved canned fish products using the CL-ELISA methods are shown in Figure 5. *A. simplex* L3 larvae were detected in all spiked products (*n* = 54) using both CL-ELISA methods. All non-spiked samples (*n* = 18) were negative in both assays. No differences in test performance were found for the examination of the following matrices: fish without additives, fish in tomato sauce, and fish in sunflower oil.

In contrast to the CL-ELISA methods, detection of *Anisakis* in autoclaved canned samples by real-time PCR failed. Results of real-time PCR were negative for all spiked and non-spiked samples.

### 2.4. Examination of Commercial Seafood Products

Results of the examination of commercial seafood products from Polish markets, details of fishing area, and place of production (if available) are shown in Table 3. As CL-S-ELISA showed the best performance, it was used for the further examination of seafood products. A total of 259 seafood products were tested, 28% of which were positive. More than half of the positive samples (*n* = 39) comprised the following smoked fish products: mackerel, herring, cod, and hake. Other positive samples were found in marinated herrings, canned cod livers, canned mackerels, and surimi sticks. No positive food samples were detected in the following species of tested products: tuna, Atlantic argentine, sardine, sprat, squid, and anchovy. The distribution of the results, depending on the species composition and type of product processing, together with statistical comparisons, are shown in Figure S1. 

## 3. Discussion

In the present study, we address two issues related to *A. simplex*: (i) we developed and validated two effective CL-ELISA methods for detecting *A. simplex* proteins in processed foods; (ii) and we used CL-S-ELISA, the assay with the highest sensitivity, to examine seafood products from Polish markets.

### 3.1. CL-immunoassays for Sensitive Detection of A. simplex L3 in Processed Food

To date, several different immunoenzymatic [16,21,22,23], molecular [24,25,26,27,28,29,30], and mass spectrometry [31,32] methods have been developed for the detection of *A simplex* in processed fish products. Nevertheless, only a few assays have been validated using samples exposed to high temperatures similar to those used in food technological processes [30,33].

In the initial stage of the study, SDS-PAGE and WB profiles of native and heated antigens were compared, and only small differences were found. Slightly higher background, lower intensity, and lower number of visible bands in the antigen profiles were probably caused by the partial degradation of antigenic proteins. However, SDS-PAGE and WB band profiles of *Anisakis* antigen after heating were clearly visible. Similar observations regarding the reduction of the number and intensity of bands in electrophoretic and WB profiles of autoclaved *Anisakis* antigen was reported in a previous study [34]. In the present investigation, we found that the heated antigen retained the ability to bind IgG antibodies and, therefore, it was possible to use them in CL-ELISA tests. The thermal resistance of *A. simplex* antigen, allergens, and the antibodies’ binding capacity of heated *Anisakis* antigens have also been confirmed in other earlier studies [7,22,35,36,37]. 

The methods developed in this study—CL-S-ELISA and CL-C-ELISA—were shown to be effective for examination of highly processed seafood products, which was confirmed in the validation process using samples spiked with *Anisakis* antigen which had been subjected to high temperatures. The practical usefulness of the assays was confirmed by examination of prepared canned and autoclaved fish products enriched with *A. simplex* larvae. The muscle tissue of cultured rainbow trout was used as a matrix for sample preparation (except for samples used to determine the specificity) used in validation, due to the lack of risk of occurrence of nematodes from the Anisakidae family.

Non-specific reactions with muscle tissue of different fish species were not found. In addition, there was no effect of commonly used additives in fish products (tomato sauce or sunflower oil) on the effectiveness of our assays. Due to the wide range of various additives used in the production of seafood products, it was not possible to include them all in the present study. Sunflower oil and tomato sauce were included in the study because there are one of the most popular additives of commercial fish products. Furthermore, sunflower oil and tomato sauce essentially differ in their chemical compositions. Sunflower oil contains mainly fats (100 g/100 mL), while carbohydrates (7.2 g/100 mL) followed by salt (1.1 g/100 mL), proteins (0.8 g/100 mL), and fats (0.6 g/100 mL) are major components of tomato sauce (according to data provided by the manufacturer). The use of additives with different compositions provided wider insights into the influence of additives on the performance of assays. Furthermore, it is important to be aware of the potential cross-reactions of anti-*A. simplex* IgG antibodies used in assays with antigens of other closely related nematodes from the Anisakidae family, such as *Anisakis pegreffii*, *Pseudoterranova* spp. and *Contracaecum* spp. Nevertheless, allergens and possible allergens were identified recently in *A. pegreffii* [38,39], *Pseudoterranova decipiens,* and *Contracaecum osculatum* [40]. Possible cross-reactivity with these anisakids does not diminish the usefulness of developed assays due to the pathogenic potential of these anisdakids. 

Previously developed sandwich ELISA and competitive ELISA techniques for the colorimetric detection of *A. simplex* in food had detection limits of 50–60 ng/g of food (in fish balls and codfish or mackerel with tomato sauce) [16] and 53 ng/mL [23], respectively. The methods developed in this study had detection limits for *A. simplex* antigens in food of 0.5 ng/mL (CL-S-ELISA) and 5 ng/mL (CL-C-ELISA). The use of chemiluminescence technology has been shown to allow for more sensitive analyte detection than a colorimetric assay by up to five orders of magnitude [41]. Indeed, this was also the case in the present study, in which a 1,2-dioxetane-based chemiluminescence substrate was used for the sensitive detection of alkaline phosphatase (AP). According to the manufacturer, this substrate has an attogram level of theoretical sensitivity of AP detection.

The protocol of sample preparation (see Section 4.7) gave the best results in both CL-ELISA tests. The phosphate-buffered saline (PBS) that was used to extract *A. simplex* proteins from the sample is often used for this purpose in many assays and is an easily accessible reagent. Accurate disintegration was a very important stage in sample preparation and, therefore, high-speed homogenization was used in combination with sonication. It should be noted that samples weighing 200 g were used in food examination tests. As *A. simplex* larvae and their fragments are generally distributed unevenly in the food product, taking low-weight samples (i.e., several grams) for ELISA, PCR, or other tests increases the risk of false negative results.

The CL-S-ELISA method developed in the present study had a better limit of detection and precision than CL-C-ELISA. The main reason for this seems to be the better suitability of polyclonal anti-*A. simplex* rabbit IgG antibodies in the sandwich ELISA system than in the competitive system. In competitive immunoassays, monoclonal antibodies are generally more effective, as they are specific for selected epitopes [42]. However, obtaining monoclonal antibodies is expensive and technically demanding. Despite the better specificity of monoclonal antibodies, polyclonal antibodies often allow for more sensitive detection of low concentration analytes [43]. Polyclonal antibodies are also preferred in immunoassays used for the examination of processed foods, as some epitopes of an analyte may be significantly altered or masked during the technological processes involved [44]. Therefore, we decided to use polyclonal antibodies in both CL-ELISA methods. Furthermore, the usefulness of polyclonal IgG antibodies in ELISA for the detection of *A. simplex* antigen has been previously demonstrated by Werner et al. [16] and Xu et al. [23].

A commercial real time-PCR test was used in this study, in order to compare the effectiveness of the example molecular assay with the developed CL-ELISA methods. Real-time PCR was not able to detect *A. simplex* in the canned products at any of the enrichment levels (1–8 larvae/200 g). According to the manufacturer’s specifications, real-time PCR should detect 0.001% of *Anisakis* spp. or *Pseudoterranova* spp. in fish DNA, and its suitability for the examination of canned products has been declared. Nevertheless, there exists no information on whether real-time PCR can detect *A. simplex* in canned fish products subjected to sterilization at 121 °C for 60 min. The poor results of the real-time PCR test were probably due to heat sterilization causing DNA fragmentation, which can have a negative impact on detection by molecular methods [45,46]. 

### 3.2. Examination of Seafood Products 

The examination of 259 seafood products allowed for a preliminary estimation of the occurrence of *A. simplex* antigens in seafood from Polish markets. Popular products that are purchased in supermarkets throughout the country were tested using CL-S-ELISA. This assay was selected due to the better performance found in the validation stage. Twenty-eight percent of examined food samples were *A. simplex* antigen positive in the CL-S-ELISA. Due to the lack of appropriate analytical methods, highly processed food products, such as canned seafood, fish salads, fish pastes, and surimi, have not been tested in Poland for *A. simplex*. Among other countries, only in Norway has a larger group of processed seafood products been tested, where 34 samples out of 130 tested positive for *A. simplex* antigens using colorimetric sandwich ELISA and mass spectrometry methods [47]. Thus, the percentage of positive food samples in Norway was similar to that in our results.

In the present study, as well as in the survey conducted by Fæste et al. [47], *A. simplex* antigen was found in products from mackerel, herring, cod, and surimi sticks; however, contrary to results from Norway, we did not detect *Anisakis* in sardine or anchovy products. In the present survey, the detection of *A. simplex* proteins in mackerel, herring, cod, and hake products was not surprising, as *Anisakis* larvae have been found in the raw fish of these species, both in studies conducted in Poland and in many other European countries [48,49,50,51]. The high prevalence and the intensity of invasion of *Anisakis* in hake and mackerel are known; therefore, these fish species considered to be “high-risk” for consumers [48,52,53]. The presence of *Anisakis* antigen in surimi sticks, as already mentioned, was found in our study and in other investigations [29,47]. The manufacturer of surimi sticks examined in the present study did not specify the species of fish used to make this product. However, it is considered that surimi sticks contain mostly hake and blue whiting [29,54,55], species of fish in which *Anisakis* spp. larvae are often found [29,51,52,53]. In the present study, *A. simplex* proteins were not detected in anchovy products. Nevertheless, larvae of *Anisakis* spp. were often detected in raw anchovies [56] and slightly processed products [57] in other studies. Furthermore, marinated anchovies are recognized as the main food vehicle of anisakiasis in Spain [58]. Anchovy products are not commonly eaten in Poland, therefore only 6 samples were tested. We did not find the *A. simplex* antigen in the sardine products. The prevalence of *Anisakis* spp. in sardines is generally quite low and also the risk of anisakiasis is at an intermediate level [52,59]. However, cases of anisakiasis and *Anisakis* allergy caused by consumption of sardines were reported [59]. In the present survey, *A. simplex* antigen was not detected in sprat products. This result is consistent with other studies of Baltic sprat [60,61] and sprat from the North Sea [62], in which a very low prevalence and intensity of Anisakidae invasion have been noted. In our study, *A. simplex* antigen was not detected in tuna and squid products either; however, it is not exactly known what species of tuna or squid were used to make these products, as this information was not provided by the manufacturer on the packaging. Detection of *Anisakis* spp. in different species of tuna [1,63,64,65] and squid [1,66,67] were reported in several studies. In the products from Atlantic argentine, *A. simplex* antigen was not found in the present survey. *Anisakis* spp. larvae were detected in the Atlantic argentine in a previous study [68]; however, there exist no large scale investigations on the occurrence of anisakids in this fish species. 

The prevalence of *Anisakis* spp. in seafood is related to fishing area. Therefore, it would be interesting to compare the occurrence of *Anisakis* antigens in food products with the corresponding fishing area. However, the food producers of many products did not specify a fishing area on the packaging, or the fishing area was not determined precisely (see Table 3). Therefore, the analysis of the occurrence of *A. simplex* antigen in food products depending on fishing area is limited. Nevertheless, according to the available data, most of the examined products were produced from fish caught in FAO area 27. *A. simplex* is a common nematode of fishes in this fishing area [2]. The general prevalence of *A. simplex* in fish, including fish of economic importance, in FAO 27 is high, up to 60% [2,69]. In fish from FAO area 27, there are also differences in the prevalence of *Anisakis* depending on the sub-area; for example, the prevalence of anisakids in herring and cod is higher in the southwest and lower in the southeast of the Baltic Sea [50,61,70].

In the present study, the majority of positive samples were smoked products; however, it is difficult to unambiguously determine the cause of this. Due to the verified ability of the CL-S-ELISA to detect *A. simplex* in heat-processed products, we think that the reason is not low sensitivity of the assay. According to EU regulations, fish products cannot contain visible parasites [14]. Thus, we suppose that, during technological processes such as filleting, highly parasitized fish are removed, but this process is limited for smoked fishes. It is worth emphasizing that the larvae of *Anisakis* spp. were found quite commonly in smoked fish in Poland [61] or other countries [71]. Except for the results of the examination of Norwegian fish products [47], there have been no other large-scale studies on highly processed products. Further studies are needed to determine the reasons for the differences in the occurrence of *Anisakis* antigen in various processed products.

## 4. Materials and Methods 

### 4.1. Ethics Statement

Rabbits purchased from the Center for Experimental Medicine (Katowice, Poland) were housed under standard conditions and experiments were conducted under the approval of the Local Ethical Commission for Animal Experimentation (licence no: 66/2012).

### 4.2. Study Workflow

**Figure 6:**
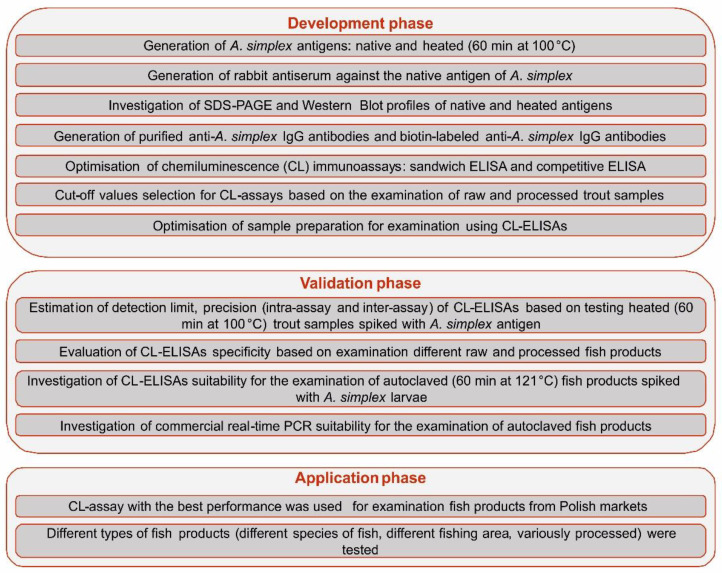
Schematic representation of study workflow.

The overall workflow of the study is shown in Figure 6.

### 4.3. A. simplex L3 Larvae Collection and Identification

*Anisakis* spp. L3 larvae were collected from marine fish as described previously [72]. Larvae were purified by washing with sterile 0.01 M PBS (pH 7.4; Sigma, St. Louis, MO, USA). The larvae were morphologically identified at the genus level using a stereomicroscope [73]. Then, species identification of *Anisakis* spp. nematodes was performed using PCR-restriction fragment length polymorphism (PCR-RFLP) [74]. Five whole larvae were used to genetic identification of *Anisakis* species.

### 4.4. Preparation of A. simplex L3 Antigens

Two different antigens of *A. simplex* were obtained:

- Native CR antigen of *A. simplex* was performed as previously reported [72];
- Heat-treated CR antigen of *A. simplex* was performed by heating native CR antigen in a thermomixer at 100 °C for 60 min.

To avoid proteolytic degradation, a protease inhibitor cocktail (Sigma, St. Louis, MO, USA) was added (100 U/mL) to antigens. Protein concentration was determined by measuring the absorbance at 280 nm using an ultraviolet-visible (UV–vis) spectrophotometer (Implen, München, Germany), adjusted to 2 mg/mL. Protein extracts were kept at −80 °C until further analysis.

### 4.5. Generation of Rabbit Anti-A. simplex Antisera

Rabbits were immunized by intramuscular injection of 2.0 mg of native *A. simplex* antigen mixed with Freund’s complete adjuvant (Sigma, St. Louis, MO, USA). Immunization was performed according to our previously described protocol [72]. Pre-immune serum was taken before immunization and used as negative control. All sera were stored at −80 °C until use.

### 4.6. Rabbit IgG Antibody Purification and Preparation of Rabbit IgG-Biotin Conjugate

IgG antibodies were affinity purified from rabbit serum immunized with *A. simplex* antigen using NAb Protein A Spin Columns (Thermo Fisher Scientific, Rockford, IL, USA) and subsequently dialyzed against PBS buffer at 4 °C in dialysis membranes (3 kDa MWCO; Thermo Fisher Scientific, Rockford, IL, USA) for 12 h. The UV-vis absorption measurement was used to determine the concentration of purified antibodies. Half batches of purified IgG antibodies were biotin labelled by EZ-Link NHS-PEO4 biotinylation kit (Thermo Fisher Scientific, Rockford, IL, USA) and dialyzed as described above. Purified IgG antibodies and biotin labelled IgG antibodies were stored at −80 °C.

### 4.7. Food Samples Preparation for ELISAs and Real-Time PCR Examinations

Two hundred grams of food sample was carefully homogenized with 600 mL PBS buffer using a high-speed homogenizer (30,000 rpm) and sonicated on ice (10 μm amplitude for 5 min). Afterward, samples were clarified by centrifugation at 5000× *g* for 20 min at 4 °C. Finally, the supernatant below the surface layer of fat was taken and stored at 4 °C for examination.

### 4.8. Types of Examined Food Samples

#### 4.8.1. Spiked and Non-Spiked Trout Samples

Food extracts were prepared from trout fillets, according to our protocol (see Section 4.7). Afterward, trout samples were used to prepare following dilutions of native *A. simplex* antigen: 0.05, 0.5, 5, 50, 100, 200, 300, and 400 ng/mL; non-spiked samples were also prepared. Seven replicates of samples with different antigen concentrations were prepared (*n* = 63). Next, samples were heated in a thermomixer at 100 °C for 60 min. The prepared samples were used for the validation tests.

#### 4.8.2. Spiked Autoclaved Canned Fish Products

Trout fillets were used to prepare three types of canned products: fish in sunflower oil, fish in tomato sauce, and fish without additives. Each canned product contained 200 g of trout muscle. Fish in sunflower oil and fish in tomato sauce were additionally enriched with 40 mL of the corresponding sauce. Fish products were spiked with 1, 4, or 8 *A. simplex* L3 larvae; non-spiked products were also prepared. Canned products were autoclaved at 120 °C for 60 min. Canned fish products at all levels of *A. simplex* spiking were prepared in 6 replicates. In total, 72 canned fish products were prepared. These samples were used to evaluate the usefulness of the CL-ELISA methods and commercial real-time PCR in examining autoclaved canned food.

#### 4.8.3. Seafood Products from the Market

Different types of seafood products (see Table 3) were obtained from markets. A total of 259 products were tested to assess the occurrence of *A. simplex* antigen in seafood products from Polish markets.

### 4.9. Sodium Dodecyl Sulfate-Polyacrylamide Gel Electrophoresis (SDS-PAGE) and Western Blot (WB) Analysis

SDS-PAGE analysis of *A. simplex* antigens and WB reactivity of antigens with rabbit anti-*A. simplex* serum were performed as described previously [40,72] using 4-chloro-1-naphthol (Sigma, St. Louis, MO, USA) as a substrate for horseradish peroxidase (HRP)-conjugated goat anti-rabbit IgG antibodies (Sigma, St. Louis, MO, USA).

The molecular weight of the SDS-PAGE and WB bands and densitometric plots of their profiles were estimated using the Bio-1D software (Vilber Lourmat, ver. 15.07, Marne-la-Vallée, France). The ImageJ software (National Institutes of Healths, ver. 1.53a, Bethesda, MD, USA) was used for densitometric calculations of the SDS-PAGE and WB profiles.

### 4.10. CL-ELISA Methods

#### 4.10.1. Materials and Reagents Used in Assays

Assays were carried out using white polystyrene high-binding microtiter strips (Lumitrac, Greiner Bio-One, Frickenhausen, Germany). Protein LoBind tubes (Eppendorf, Hamburg, Germany) were used to make dilutions of assay reagents. The coating buffer used was 0.05 M carbonate-bicarbonate buffer (pH 9.6; Sigma, St. Louis, MO, USA). The blocking buffer was 1% biotin-free casein in Tris-buffered saline (TBS, pH 7.4; Bio-Rad, Hercules, CA, USA), while the incubation buffer for biotin-labelled rabbit IgG antibodies and streptavidin-labeled AP conjugate (Sigma, St. Louis, MO, USA) was 1% biotin-free casein in TBS (same as blocking buffer) supplemented with 0.1% of polyoxyethylene (20) sorbitan monolaurate (Tween-20; Sigma, St. Louis, MO, USA). Washing buffer was 0.01 M TBS containing 0.1% Tween-20 (TBST). The chemiluminescent substrate was Chemiluminescent AP select Plus 450 (Neogen, Lexington, KY, USA) and signal intensity values were recorded in relative light units (RLU) using a Luminoskan Ascent luminometer (Thermo Fisher Scientific, Waltham, MA, USA). The optimal concentrations of antibodies, antigen, conjugate, and sample dilution used in the CL-ELISA methods were established by checker-board titration.

#### 4.10.2. Sandwich CL-ELISA (S-CL-ELISA) Protocol

The capture antibody was coated by adding 100 μL of 1 μg/mL anti-rabbit IgG antibodies to each strip well and incubating at 4 °C overnight. The strips were then washed 3 times with TBST and blocked by adding 200 μL of blocking buffer for 1 h at 37 °C. Next, strips were washed 3 times with TBST and 100 μL of samples was added to each well and incubated overnight at 4 °C with gentle shaking. After washing of the strips as described above, 100 μL of 0.05 μg/mL biotinylated rabbit anti-*Anisakis* IgG antibodies diluted in incubation buffer was added to each well and incubated at 37 °C for 1 h with gentle shaking. Subsequently, strips were washed 3 times with washing buffer, and 100 μL of 0.005 μg/mL conjugate was added to each well and incubated at 37 °C for 30 min with gentle shaking. After washing strips with TBST 3 times, 50 μL of substrate was added to each well and strips were gently shaken for 60 min at room temperature. Finally, RLU values were measured using a luminometer.

#### 4.10.3. Competitive CL-ELISA (C-CL-ELISA) Protocol

Each well of microtiter strips was coated with 100 μL of 0.5 μg/mL CR *A. simplex* antigen and incubated at 4 °C overnight. Simultaneously, 500 μL of samples were pre-incubated with 100 μL of 0.1 μg/mL biotinylated rabbit anti-*A. simplex* IgG antibodies in 1 mL tubes, at 4 °C on a rotator overnight. After overnight incubation, the strips were washed 3 times with TBST and blocked with 200 μL of blocking buffer for 1 h at 37 °C. Next, wells were washed as described above and 100 μL of pre-incubated samples were transferred to wells of strips and incubated for 1 h at 37 °C with gentle shaking. After three times washing of strips with TBST, 100 μL of 0.005 μg/mL conjugate diluted in incubation buffer was added to each well and incubated at 37 °C for 30 min with gentle shaking. Afterward, wells were washed three times with washing buffer and 50 μL of substrate was added to each well and strips were gently shaken for 60 min at room temperature. RLU values were measured using a luminometer.

#### 4.10.4. Selection of Cut-Off Values

The cut-off values for the CL-ELISA methods were established from the RLU observed in immunoassays of the following 30 samples: raw trout fillets (*n* = 10), smoked trouts (*n* =10), canned trouts in tomato sauce (*n* = 5), and canned trout in sunflower oil (*n* = 5). Mean RLU +/− 3 times standard deviation of the samples was used as the cut-off. Additionally, the suitability of calculated cut-off values was confirmed by receiver operator characteristic (ROC) curve analysis using the MedCalc software (MedCalc Software Ltd., ver. 19.3, Mariakerke, Belgium).

#### 4.10.5. Validation of Assays

The specificity of the optimized assays was evaluated by measuring the cross-reactivity with samples of fish fillets of the following fish species: trout, cod, herring, mackerel, and salmon. Three samples were tested for each fish species (*n* = 15). The absence of *Anisakis* spp. larvae in the tested fillets was confirmed by the pressing method. Furthermore, the following processed fish products were also used for the specificity determination of tests: smoked trout, canned trout, canned trout in sunflower oil, and canned trout in tomato sauce. Three samples were examined for each fish product (*n* = 12). 

The limit of detection (LOD) was calculated as the lowest concentration of *A. simplex* CR antigen that could be distinguished from a sample containing no analyte. Trout samples spiked with *Anisakis* antigen extracts (*n* = 63; see Section 4.8.1) and heated 60 min at 100 °C were examined to evaluate the LOD of ELISAs. 

Spiked and heated extracts of trout fillets that contained *A. simplex* antigen in a concentration equal or higher than LOD of the assay were used to evaluate the intra- and inter-assay variability (precision), which are represented by the coefficient of variation (CV%). The intra-assay CV% was measured by analyzing 5 replicates of each sample in the assay. The inter-assay CV% was calculated by analyzing 5 replicates of each sample carried out on 3 different days.

#### 4.10.6. Data Analysis

The Medcalc software (MedCalc Software Ltd, ver. 19.3, Mariakerke, Belgium) was used to perform the McNemar *χ*^2^ test, ROC analysis, and calculate the area under the ROC curve (AUC). The four-parameter logistic regression curve-fitting and coefficient of determination (R^2^) were performed using the GraphPad Prism software (GraphPad Software, ver. 8.3.1, La Jolla, CA, USA). The standard deviations and coefficient of variations (CV%) were calculated using Microsoft Excel 2016 (Microsoft, Redmond, WA, USA). These analyses were performed to evaluate and compare the performances of CL-S-ELISA and CL-C-ELISA. 

Two-tailed Fisher’s exact test (*p* < 0.05) and 95% confidence intervals (95% CI) were calculated to analyse the differences in *A. simplex* antigen detection in different seafood products. Calculations were performed using an online tool, Graphpad QuickCalcs (https://www.graphpad.com/quickcalcs/). 

### 4.11. Commercial Real-Time PCR Assay

Spiked sterilized canned fish products (see Section 4.8.2) were prepared according to our protocol (see Section 4.7) and samples were subsequently subjected to DNA extraction using a DNeasy Blood & Tissue Kit (Qiagen, Hilden, Germany), as per the manufacturer’s instructions. Anisakids PCR Real Time kit (4Lab Diagnostics, Casalmaggiore, Italy) was used to examine samples, according to the manufacturer’s instructions. Real-time PCR assays were performed using the Bio-Rad CFX-96 system (Bio-Rad, Hercules, CA, USA). 

## 5. Conclusions

The present study provided (i) novel, highly sensitive CL-ELISA methods for the detection of *A. simplex* antigen in processed seafood, and (ii) a preliminary assessment of *A. simplex* antigen occurrences in seafood products from Polish markets.

Both developed CL-ELISA methods were sufficiently sensitive for the examination of heat-processed fish products; however, CL-S-ELISA had better performance than CL-C-ELISA. The results of our investigations showed that CL-immunoassays are highly effective tools for *A. simplex* antigen detection, which is useful for food control laboratories.

*A. simplex* antigen was detected in 72 out of 259 popular seafood products from Polish markets using CL-S-ELISA. Based on these findings, it can be concluded that around 28% of processed seafood products in Polish markets contain *A. simplex* antigen and, therefore, may pose potential allergic hazards for sensitized consumers. Further studies are necessary to estimate the occurrence of *A. simplex* proteins in a larger group of different seafood products.

## Figures and Tables

**Table 1:** Overall densitometric measurements of SDS-PAGE and Western Blot (WB) profiles of native and heated (for 60 min at 100 °C) CR antigens of *A. simplex*. Values were calculated using the ImageJ software (National Institutes of Healths, ver. 1.53a, Bethesda, MD, USA).

	Mean Gray Value ^5^	SD of the Gray Values ^6^	Min Gray Value ^7^	Max Gray Value ^8^	Integrated Density ^9^
SDS-PAGE Native ^1^	184.8	42	56	255	50,513,856
SDS-PAGE Heated ^2^	184.93	35.23	102	254	49,073,619
WB Native ^3^	203.76	34.69	74	253	55,856,228
WB Heated ^4^	204	29.27	131	253	55,923,273

Densitometric measurements of SDS-PAGE profiles of native ^1^ and heated ^2^ antigens of *A. simplex*; densitometric measurements of WB profiles of native ^3^ and heated ^4^ antigens of *A. simplex*; mean gray value is the sum of the gray values of all the pixels in the profile divided by the number of pixels ^5^; standard deviation (SD) of the gray values used to generate the mean gray value ^6^; minimum ^7^ and maximum ^8^ gray values within the profile; integrated density is the sum of the values of the pixels in the profile ^9^.

**Table 2:** Limit of detection and precision of chemiluminescence immunoassays.

Validation Parameter	CL-S-ELISA ^1^	CL-C-ELISA ^1^
Limit of detection (ng/mL)	0.5	5
Intra-assay precision (CV% ^2^)	1.5–4.3	5.1–7.5
Inter-assay precision (CV%)	2.7–7.7	6.2–13.5

^1^ The limit of detection, intra-assay precision, and inter-assay precision were calculated using trout samples non-spiked and spiked with A. simplex antigen, which were subsequently heated for 60 min at 100 °C; ^2^ CV%, percent coefficient of variation.

**Table 3:** Results of the examination of seafood products from Polish markets. CL-S-ELISA was used for the detection of *A. simplex* antigen in products.

Seafood Product	Fishing Area ^1^	Processing Place ^1^	No. of Tested	No. Positive
Carcass of smoked mackerel	North Sea	Poland	10	9
Carcass of smoked Atlantic argentine	- ^2^	-	8	0
Buckling	-	-	9	9
Carcass of smoked herring	-	-	10	7
Smoked black cod	-	-	8	6
Carcass of smoked hake	-	-	10	8
Surimi sticks	-	Lithuania	7	7
Tuna paste	-	Poland	9	0
Mackerel paste	-	Poland	5	0
Mackerel paste	-	Sweden	5	0
Herring fillets a’la matjas	Northeast Atlantic, North Sea, or Norwegian Sea	Poland	10	8
Canned cod liver in own fat	Atlantic	Norway	8	8
Canned grilled mackerel fillets with extra virgin olive oil	Northeast Atlantic or Middle East Atlantic	Poland	9	3
Canned mackerel fillets in oil	Northeast Atlantic or Middle East Atlantic	Poland	10	3
Canned mackerel fillets in tomato sauce	Northeast Atlantic	Poland	10	4
Canned sardines in own sauce	Baltic Sea	Poland	10	0
Canned sprats in tomato sauce	Northwest Atlantic	Poland	10	0
Canned tuna chunks in gravy	Pacific	Poland	8	0
Canned spicy mackerel salad	Northeast Atlantic	Poland	10	0
Canned minced sprat with rice, vegetable paste, and spices	Baltic Sea	Poland	10	0
Canned sprat in oil	Baltic Sea	Poland	9	0
Fried pieces of Atlantic argentine in letscho	-	Poland	8	0
Canned Squid in garlic sauce with sunflower	-	Spain	10	0
Canned tuna with vegetables	Pacific	Poland	10	0
Pieces of fried cod in vegetable and tomato sauce	-	Poland	10	0
Cooked mackerel fillets in aspic	-	Poland	10	0
Anchovies in sunflower oil	-	Morocco	6	0
Filets of marinated herring with tomato paste	Baltic Sea	Poland	10	0
Herring fillets in cream sauce	Skagerrak and Kattegat or North Sea	Poland	10	0
**Total no.**			**259**	**72**

^1^ Fishing area and processing place provided on the packaging; ^2^ -, data not provided on the packaging of a product.

## Supplementary Materials

The following are available online at https://www.mdpi.com/2076-0817/9/10/777/s1, File S1: ROC curve analysis for evaluating the performance of CL-S-ELISA and CL-C-ELISA. Figure S1: Bar charts showing the percentage of positive samples depending on the species composition. (a) and type of product processing (b). The bar errors show 95% confidence intervals. The tables show statistical comparison results using Fisher’s exact test with Bonferroni correction. Statistically significant differences are highlighted in blue.
