# Supplementary material for: Development and Application of Novel Chemiluminescence Immunoassays for Highly Sensitive Detection of Anisakis simplex Proteins in Thermally Processed Seafood
Source: Pathogens. 2020 Sep 23;9(10):777. doi: 10.3390/pathogens9100777 (PMC7598195; doi:10.3390/pathogens9100777)
Supplement: Supplementary file 1 [file pathogens-09-00777-s001.zip › Supplementary Material/Supplemental File S1.pdf]

ROC curve CL-C-ELISA

Variable	RLU
Classification variable	Result

Sample size	63
Positive group [a]	56 (88,89%)
Negative group [b]	7 (11,11%)

[a] Result = 1
[b] Result = 0

Area under the ROC curve (AUC)

Area under the ROC curve (AUC)	0,918
Standard Error [a]	0,0353
95% Confidence interval [b]	0,821 to 0,972
95% Bootstrap CI [c]	0,824 to 0,969
z statistic	11,867
Significance level P (Area=0.5)	<0,0001

[a] DeLong et al., 1988
[b] Binomial exact
[c] BC_a bootstrap confidence interval (1000 iterations; random number seed: 978).

Youden index

Youden index J	0,8571
95% Confidence interval [a]	0,7321 to 0,9286
Associated criterion	≤427
95% Confidence interval [a]	≤426 to ≤441
Sensitivity	85,71
Specificity	100,00

[a] BC_a bootstrap confidence interval (1000 iterations; random number seed: 978).

Summary Table

Estimated specificity at fixed sensitivity			
Sensitivity	Specificity	95% CI [a]	Criterion
80,00	100,00	71,43 to 100,00	≤414,4
90,00	65,71	0,00 to 100,00	≤430,4
95,00	40,00	0,00 to 100,00	≤442,2
97,50	0,00	0,00 to 34,29	≤451,2
99,00	0,00	0,00 to 42,86	≤454,64
**Estimated sensitivity at fixed specificity**			
Specificity	Sensitivity	95% CI [a]	Criterion
80,00	85,71	71,43 to 92,86	≤427,7
90,00	85,71	73,21 to 92,86	≤427,35
95,00	85,71	73,21 to 92,86	≤427,175
97,50	85,71	0,00 to 0,00	≤427,0875
99,00	85,71	0,00 to 0,00	≤427,035

[a] BC_a bootstrap confidence interval (1000 iterations; random number seed: 978).

Criterion values and coordinates of the ROC curve [Hide]

Criterion	Sensitivity	95% CI	Specificity	95% CI	+LR	-LR
<42	0,00	0,0 - 6,4	100,00	59,0 - 100,0		1,00
≤42	1,79	0,05 - 9,6	100,00	59,0 - 100,0		0,98
≤43	3,57	0,4 - 12,3	100,00	59,0 - 100,0		0,96
≤46	7,14	2,0 - 17,3	100,00	59,0 - 100,0		0,93
≤47	8,93	3,0 - 19,6	100,00	59,0 - 100,0		0,91
≤48	10,71	4,0 - 21,9	100,00	59,0 - 100,0		0,89
≤49	12,50	5,2 - 24,1	100,00	59,0 - 100,0		0,88
≤55	14,29	6,4 - 26,2	100,00	59,0 - 100,0		0,86
≤58	17,86	8,9 - 30,4	100,00	59,0 - 100,0		0,82
≤59	19,64	10,2 - 32,4	100,00	59,0 - 100,0		0,80
≤61	21,43	11,6 - 34,4	100,00	59,0 - 100,0		0,79
≤63	23,21	13,0 - 36,4	100,00	59,0 - 100,0		0,77
≤68	25,00	14,4 - 38,4	100,00	59,0 - 100,0		0,75
≤71	26,79	15,8 - 40,3	100,00	59,0 - 100,0		0,73
≤75	28,57	17,3 - 42,2	100,00	59,0 - 100,0		0,71
≤77	30,36	18,8 - 44,1	100,00	59,0 - 100,0		0,70
≤79	33,93	21,8 - 47,8	100,00	59,0 - 100,0		0,66
≤82	35,71	23,4 - 49,6	100,00	59,0 - 100,0		0,64
≤86	37,50	24,9 - 51,5	100,00	59,0 - 100,0		0,63
≤108	39,29	26,5 - 53,2	100,00	59,0 - 100,0		0,61
≤109	41,07	28,1 - 55,0	100,00	59,0 - 100,0		0,59
≤112	42,86	29,7 - 56,8	100,00	59,0 - 100,0		0,57
≤114	44,64	31,3 - 58,5	100,00	59,0 - 100,0		0,55
≤119	46,43	33,0 - 60,3	100,00	59,0 - 100,0		0,54
≤125	48,21	34,7 - 62,0	100,00	59,0 - 100,0		0,52
≤129	50,00	36,3 - 63,7	100,00	59,0 - 100,0		0,50
≤155	51,79	38,0 - 65,3	100,00	59,0 - 100,0		0,48
≤158	53,57	39,7 - 67,0	100,00	59,0 - 100,0		0,46
≤165	55,36	41,5 - 68,7	100,00	59,0 - 100,0		0,45
≤172	57,14	43,2 - 70,3	100,00	59,0 - 100,0		0,43
≤179	58,93	45,0 - 71,9	100,00	59,0 - 100,0		0,41
≤181	60,71	46,8 - 73,5	100,00	59,0 - 100,0		0,39
≤189	62,50	48,5 - 75,1	100,00	59,0 - 100,0		0,38
≤235	64,29	50,4 - 76,6	100,00	59,0 - 100,0		0,36
≤251	66,07	52,2 - 78,2	100,00	59,0 - 100,0		0,34
≤262	67,86	54,0 - 79,7	100,00	59,0 - 100,0		0,32
≤270	69,64	55,9 - 81,2	100,00	59,0 - 100,0		0,30
≤285	71,43	57,8 - 82,7	100,00	59,0 - 100,0		0,29
≤289	73,21	59,7 - 84,2	100,00	59,0 - 100,0		0,27
≤295	75,00	61,6 - 85,6	100,00	59,0 - 100,0		0,25
≤407	76,79	63,6 - 87,0	100,00	59,0 - 100,0		0,23
≤412	78,57	65,6 - 88,4	100,00	59,0 - 100,0		0,21
≤415	80,36	67,6 - 89,8	100,00	59,0 - 100,0		0,20
≤417	82,14	69,6 - 91,1	100,00	59,0 - 100,0		0,18

≤426	83,93	71,7 - 92,4	100,00	59,0 - 100,0		0,16
≤427	85,71	73,8 - 93,6	100,00	59,0 - 100,0		0,14
≤428	85,71	73,8 - 93,6	71,43	29,0 - 96,3	3,00	0,20
≤429	87,50	75,9 - 94,8	71,43	29,0 - 96,3	3,06	0,18
≤430	89,29	78,1 - 96,0	71,43	29,0 - 96,3	3,13	0,15
≤431	91,07	80,4 - 97,0	57,14	18,4 - 90,1	2,12	0,16
≤432	92,86	82,7 - 98,0	57,14	18,4 - 90,1	2,17	0,13
≤434	92,86	82,7 - 98,0	42,86	9,9 - 81,6	1,63	0,17
≤441	94,64	85,1 - 98,9	42,86	9,9 - 81,6	1,66	0,13
≤447	96,43	87,7 - 99,6	28,57	3,7 - 71,0	1,35	0,13
≤449	96,43	87,7 - 99,6	14,29	0,4 - 57,9	1,12	0,25
≤450	96,43	87,7 - 99,6	0,00	0,0 - 41,0	0,96	
≤452	98,21	90,4 - 100,0	0,00	0,0 - 41,0	0,98	
≤458	100,00	93,6 - 100,0	0,00	0,0 - 41,0	1,00	

ROC curve CL-S-ELISA

Variable	RLU
Classification variable	Result

Sample size	63
Positive group [a]	56 (88,89%)
Negative group [b]	7 (11,11%)

[a] Result = 1
[b] Result = 0

Area under the ROC curve (AUC)

Area under the ROC curve (AUC)	0,943
Standard Error [a]	0,0284
95% Confidence interval [b]	0,854 to 0,986
95% Bootstrap CI [c]	0,861 to 0,982
z statistic	15,604
Significance level P (Area=0.5)	<0,0001

[a] DeLong et al., 1988
[b] Binomial exact
[c] BC_a bootstrap confidence interval (1000 iterations; random number seed: 978).

Youden index

Youden index J	0,8750
95% Confidence interval [a]	0,7500 to 0,9464
Associated criterion	>34
95% Confidence interval [a]	>33 to >34
Sensitivity	87,50
Specificity	100,00

[a] BC_a bootstrap confidence interval (1000 iterations; random number seed: 978).

Summary Table

Estimated specificity at fixed sensitivity			
Sensitivity	Specificity	95% CI [a]	Criterion
80,00	100,00	91,92 to 100,00	>49,2
90,00	80,00	17,14 to 100,00	>32,6
95,00	50,48	3,23 to 100,00	>31,266666667
97,50	25,71	0,00 to 60,00	>30,4
99,00	14,29	0,00 to 53,75	>30
Estimated sensitivity at fixed specificity			
Specificity	Sensitivity	95% CI [a]	Criterion
80,00	90,00	78,47 to 96,43	>32,6
90,00	88,75	76,78 to 95,36	>33,3
95,00	88,13	76,94 to 95,27	>33,65
97,50	87,81	0,00 to 0,00	>33,825
99,00	87,62	0,00 to 0,00	>33,93

[a] BC_a bootstrap confidence interval (1000 iterations; random number seed: 978).

Criterion values and coordinates of the ROC curve [Hide]

Criterion	Sensitivity	95% CI	Specificity	95% CI	+LR	-LR
≥30	100,00	93,6 - 100,0	0,00	0,0 - 41,0	1,00	
>30	98,21	90,4 - 100,0	14,29	0,4 - 57,9	1,15	0,13
>31	96,43	87,7 - 99,6	42,86	9,9 - 81,6	1,69	0,083
>32	91,07	80,4 - 97,0	71,43	29,0 - 96,3	3,19	0,13
>33	89,29	78,1 - 96,0	85,71	42,1 - 99,6	6,25	0,13
>34	87,50	75,9 - 94,8	100,00	59,0 - 100,0		0,13
>47	85,71	73,8 - 93,6	100,00	59,0 - 100,0		0,14
>48	83,93	71,7 - 92,4	100,00	59,0 - 100,0		0,16
>49	80,36	67,6 - 89,8	100,00	59,0 - 100,0		0,20
>50	78,57	65,6 - 88,4	100,00	59,0 - 100,0		0,21
>51	76,79	63,6 - 87,0	100,00	59,0 - 100,0		0,23
>54	75,00	61,6 - 85,6	100,00	59,0 - 100,0		0,25
>255	73,21	59,7 - 84,2	100,00	59,0 - 100,0		0,27
>260	71,43	57,8 - 82,7	100,00	59,0 - 100,0		0,29
>263	69,64	55,9 - 81,2	100,00	59,0 - 100,0		0,30
>269	67,86	54,0 - 79,7	100,00	59,0 - 100,0		0,32
>278	66,07	52,2 - 78,2	100,00	59,0 - 100,0		0,34
>281	64,29	50,4 - 76,6	100,00	59,0 - 100,0		0,36
>288	62,50	48,5 - 75,1	100,00	59,0 - 100,0		0,38
>576	60,71	46,8 - 73,5	100,00	59,0 - 100,0		0,39
>586	58,93	45,0 - 71,9	100,00	59,0 - 100,0		0,41
>591	57,14	43,2 - 70,3	100,00	59,0 - 100,0		0,43
>608	55,36	41,5 - 68,7	100,00	59,0 - 100,0		0,45
>610	53,57	39,7 - 67,0	100,00	59,0 - 100,0		0,46
>615	51,79	38,0 - 65,3	100,00	59,0 - 100,0		0,48
>622	50,00	36,3 - 63,7	100,00	59,0 - 100,0		0,50
>923	48,21	34,7 - 62,0	100,00	59,0 - 100,0		0,52
>953	46,43	33,0 - 60,3	100,00	59,0 - 100,0		0,54
>967	44,64	31,3 - 58,5	100,00	59,0 - 100,0		0,55
>970	42,86	29,7 - 56,8	100,00	59,0 - 100,0		0,57
>973	41,07	28,1 - 55,0	100,00	59,0 - 100,0		0,59
>987	39,29	26,5 - 53,2	100,00	59,0 - 100,0		0,61
>994	37,50	24,9 - 51,5	100,00	59,0 - 100,0		0,63
>1431	35,71	23,4 - 49,6	100,00	59,0 - 100,0		0,64
>1463	33,93	21,8 - 47,8	100,00	59,0 - 100,0		0,66
>1478	32,14	20,3 - 46,0	100,00	59,0 - 100,0		0,68
>1484	30,36	18,8 - 44,1	100,00	59,0 - 100,0		0,70
>1486	28,57	17,3 - 42,2	100,00	59,0 - 100,0		0,71
>1499	26,79	15,8 - 40,3	100,00	59,0 - 100,0		0,73
>1535	25,00	14,4 - 38,4	100,00	59,0 - 100,0		0,75
>1910	23,21	13,0 - 36,4	100,00	59,0 - 100,0		0,77
>1921	21,43	11,6 - 34,4	100,00	59,0 - 100,0		0,79
>1927	19,64	10,2 - 32,4	100,00	59,0 - 100,0		0,80
>1956	17,86	8,9 - 30,4	100,00	59,0 - 100,0		0,82

>1974	16,07	7,6 - 28,3	100,00	59,0 - 100,0		0,84
>1986	14,29	6,4 - 26,2	100,00	59,0 - 100,0		0,86
>1988	12,50	5,2 - 24,1	100,00	59,0 - 100,0		0,88
>2185	10,71	4,0 - 21,9	100,00	59,0 - 100,0		0,89
>2198	8,93	3,0 - 19,6	100,00	59,0 - 100,0		0,91
>2241	7,14	2,0 - 17,3	100,00	59,0 - 100,0		0,93
>2256	5,36	1,1 - 14,9	100,00	59,0 - 100,0		0,95
>2259	3,57	0,4 - 12,3	100,00	59,0 - 100,0		0,96
>2270	1,79	0,05 - 9,6	100,00	59,0 - 100,0		0,98
>2285	0,00	0,0 - 6,4	100,00	59,0 - 100,0		1,00
