# Supplementary figures and images for: Development and Application of Novel Chemiluminescence Immunoassays for Highly Sensitive Detection of Anisakis simplex Proteins in Thermally Processed Seafood
Source: Pathogens. 2020 Sep 23;9(10):777. doi: 10.3390/pathogens9100777 (PMC7598195; doi:10.3390/pathogens9100777)

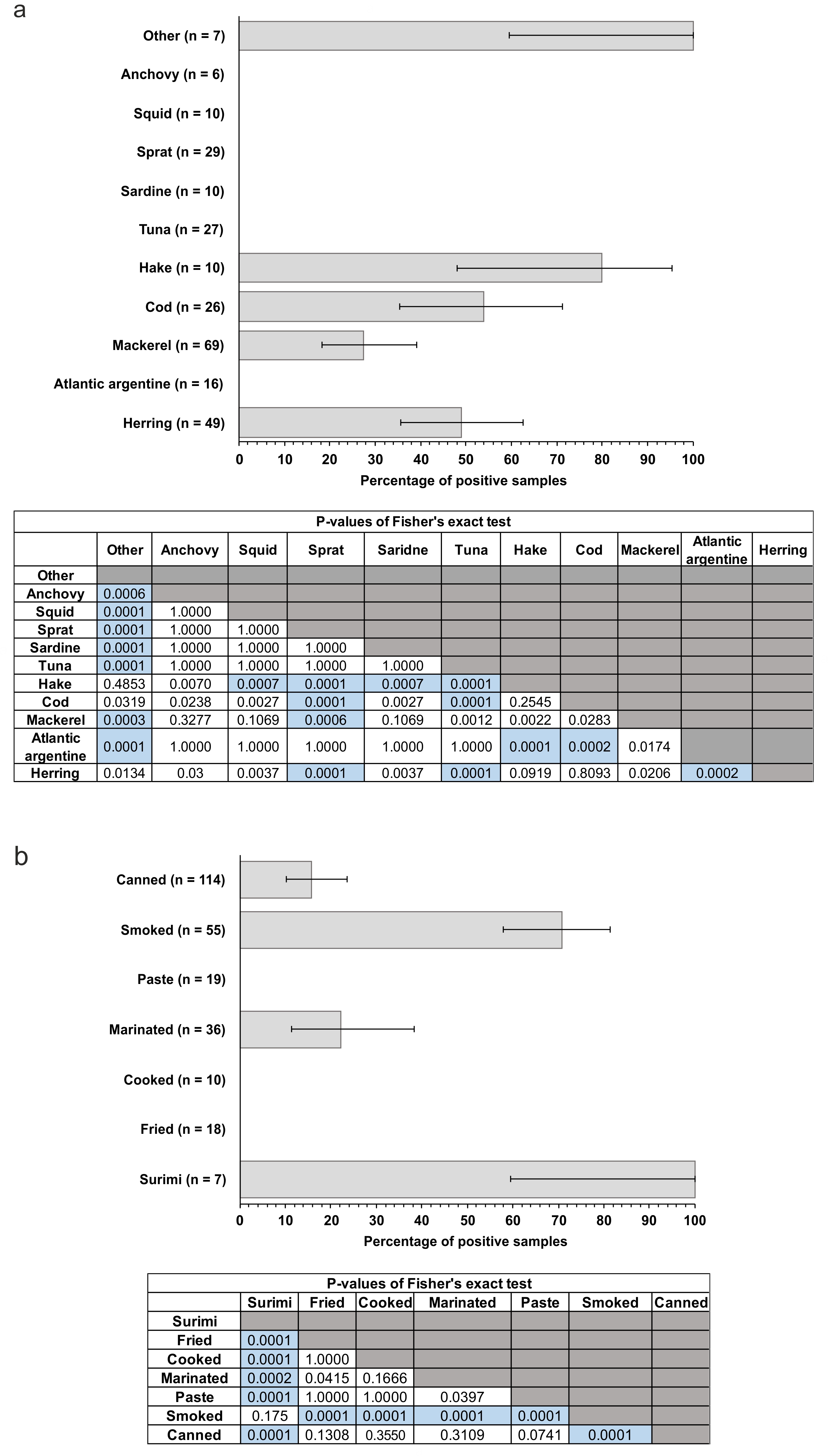

Supplement: Supplementary file 1 [file pathogens-09-00777-s001.zip › Supplementary Material/Supplemental Figure S1.jpg]
